# Reduced aqueous humour ascorbic-acid concentration in women with smaller anterior chamber depth

**DOI:** 10.1038/s41598-018-36899-8

**Published:** 2019-01-23

**Authors:** Sakae Ito, Toshimi Sairenchi, Takehisa Machida, Yuka Takino, Yoshitaka Kondo, Koichiro Mukai, Gen Kobashi, Akihito Ishigami, Tadashi Senoo

**Affiliations:** 1 Department of Ophthalmology, Dokkyo Medical University, 880 Kitakobayashi, Shimotsuga-gun, Tochigi 321-0293 Japan; 2 Molecular Regulation of Aging, Tokyo Metropolitan Institute of Gerontology, 35-2 Sakae-cho, Itabashi-ku, Tokyo 173-0015 Japan; 3 Department of Ophthalmology, Nogi Hospital, 5320-2 Tomonuma, Shimotsuga-gun, Tochigi 329-0101 Japan; 4 Department of public health, Dokkyo Medical University, 880 Kitakobayashi, Shimotsuga-gun, Tochigi 321-0293 Japan

## Abstract

Short anterior chamber depth (ACD) is considered a risk factor of endothelial-cell loss after phacoemulsification. However, whether it is an independent risk factor or not remains controversial. We investigated the relationship between ascorbic acid (AA) concentrations in the aqueous humour (AqH) and ACD. We analysed 165 AqH samples of 97 patients (42 men and 55 women) who underwent small incision cataract surgery. AqH and plasma AA concentrations were measured using a high-performance liquid chromatography - electrochemical detection method. Patient characteristics were compared between and within the sexes. As a result, age and ACD were significantly correlated with AqH AA concentrations (r = −0.206, P = 0.045; r = 0.339, P < 0.001) only in women. Moreover, plasma AA concentrations were significantly correlated with AqH AA concentrations (r = 0.420, P < 0.001; r = 0.316, P = 0.002) both in men and women. After adjusting for confounding factors (age and plasma AA concentrations), ACD was significantly and positively correlated with AqH AA concentrations (partial.r = 0.275, P = 0.009) only in women. In conclusion, AqH AA concentrations were reduced in women with smaller ACD. This may suggest that women with short ACD could be more susceptible to oxidative damage.

## Introduction

Ascorbic acid (AA) concentration in the human aqueous humour (AqH) is more than 20-fold higher than in the plasma^1–3^. There are various theories regarding the reasons why AqH AA concentration is high. First, it has been suggested that AA acts as an ultraviolet filter for internal eye structures because diurnal mammals have higher AqH AA concentrations than nocturnal mammals^4^. AA absorbs ultraviolet light of 310 nm or less and reduces fluorescence emission of ultraviolet A of 320 to 400 nm^5,6^. Second, AA controls the metabolism of the extracellular matrix of tissues that are in contact with the AqH because AA regulates the synthesis of various extracellular-matrix molecules such as collagen and elastin^7,8^. In addition, AA is considered a radical scavenger in the eye. Free radical species *in vivo* reacts with stable molecules such as nucleic acids, proteins, sugars, and lipids and promotes oxidisation, which results in various disease states. AA has strong reducing action and protects the cornea, crystalline lens, and other intraocular tissues from oxidative damage^9–12^.

Ultrasonic phacoemulsification for cataract surgery results in the formation of free radical species and causes injury in corneal endothelial cells^13,14^. In contrast, adding the antioxidant AA to the irrigation solution significantly reduces corneal endothelial-cell damage^10,12^. The risk of endothelial-cell loss after phacoemulsification depends on several preoperative and intraoperative parameters (high nucleus grade, advanced age, long phaco time, high ultrasound energy, short axial length, and surgical skill)^15–18^. Especially, Walkow *et al*.^15^ and Storr-Paulsen *et al*.^16^ reported that eyes with shorter axial length (AL) had significantly increased risk for endothelial-cell loss. Although the direct relationship between anterior chamber depth (ACD) and endothelial-cell loss remains unclear, strong positive correlations between ACD and AL have been frequently reported^19–21^. Some researchers have considered that short ACD might be a risk factor for corneal endothelial-cell damage^15,22,23^. Short ACD leads to phacoemulsification being performed closer to the corneal endothelial cells and may therefore be associated with an increased risk of corneal endothelial-cell loss. However, to the best of our knowledge, no report has considered the association between ACD and AA concentrations in the AqH. We hypothesised that short ACD would be more susceptible to oxidative damage and that it would be associated with decrease in AqH AA concentration. Therefore, in this study, we examined the relationship between ACD and AqH AA concentrations in patients with cataract and examined whether the association between these two factors is affected by patient characteristics.

## Results

### Patient characteristics

Patient characteristics are presented in Table 1. Seventy eyes of 42 men and 95 eyes of 55 women were included. ACD and AL were significantly shorter in women (3.34 ± 0.37 mm vs. 3.09 ± 0.42 mm; P < 0.001, 24.0 ± 1.3 mm vs. 23.5 ± 1.5 mm; P = 0.042, respectively). AqH AA concentrations were significantly lower in men (1535 ± 326 μmol/L vs. 1733 ± 355 μmol/L, respectively; P < 0.001), whereas there was no significant difference in plasma AA concentrations between the sexes (48.4 ± 18.1 μmol/L vs. 52.7 ± 15.9 μmol/L, respectively; P = 0.105).

**Table 1:** Patient characteristics by sex.

Parameter	Men	Women	p-value
	n = 42	n = 55	
No. of eyes	70	95	
Age (years)	74.4 ± 7.7	75.6 ± 8.3	0.363*
UCVA (logMAR)	0.69 ± 0.45	0.71 ± 0.47	0.814*
BCVA (logMAR)	0.33 ± 0.40	0.34 ± 0.37	0.832*
IOP (mmHg)	12.7 ± 3.1	13.4 ± 2.8	0.163*
NS (Emery-Little classification)	2.4 ± 0.7	2.3 ± 0.9	0.371^†^
ECD (cells/mm^2^)	2624 ± 325	2549 ± 317	0.138*
ACD (mm)	3.34 ± 0.37	3.09 ± 0.42	<0.001*
AL (mm)	24.0 ± 1.3	23.5 ± 1.5	0.042*
CCT (μm)	530 ± 53	529 ± 29	0.836*
AqH AA concentrations (μmol/L)	1535 ± 326	1733 ± 355	<0.001*
Plasma AA concentrations (μmol/L)	48.4 ± 18.1	52.7 ± 15.9	0.105*

Data in columns are mean ± SD. UCVA, uncorrected visual acuity; BCVA, best-corrected visual acuity; IOP, intraocular pressure; NS, nuclear sclerosis; ECD, endothelial-cell density; ACD, anterior chamber depth; AL, axial length; CCT, central corneal thickness; AqH, aqueous humour; AA, ascorbic acid ^*^Student’s t-tests. ^†^Mann-Whitney U test.

### Correlation between AqH AA concentrations and patient characteristics

The sex-specific correlation coefficients between ACD and the characteristics of the patients are shown in Table 2. The AqH AA concentrations were significantly correlated with age (r = −0.206, P = 0.045), ACD (r = 0.339, P < 0.001) and plasma AA concentrations (r = 0.316, P = 0.002) in women. The AqH AA concentrations were significantly correlated with plasma AA concentrations (r = 0.420, P < 0.001) in men. AqH AA was not significantly associated with nuclear sclerosis (NS), endothelial-cell density (ECD), AL, or central corneal thickness (CCT).

**Table 2:** Correlation between AqH AA concentrations and patient characteristics.

Factor	Men		Women	
	r	p-value	r	p-value
Age	−0.096	0.429*	−0.206	0.045*
UCVA	0.046	0.707*	0.004	0.972*
BCVA	0.173	0.151*	−0.065	0.530*
IOP	−0.230	0.055*	−0.095	0.361*
NS	−0.027	0.821^†^	−0.144	0.164^†^
ECD	0.058	0.635*	0.048	0.647*
ACD	−0.043	0.728*	0.339	<0.001*
AL	0.045	0.714*	0.151	0.144*
CCT	−0.093	0.287*	0.150	0.153*
Plasma AA concentrations	0.420	< 0.001*	0.316	0.002*

UCVA, uncorrected visual acuity; BCVA, best-corrected visual acuity; IOP, intraocular pressure; NS, nuclear sclerosis; ECD, endothelial-cell density; ACD, anterior chamber depth; AL, axial length; CCT, central corneal thickness; AA, ascorbic acid; r, correlation coefficient. *Pearson’s correlation coefficient. ^†^Spearman’s correlation coefficient.

### Correlations between AL and AqH AA concentrations

Table 3 shows the sex-specific correlation coefficients between AL and AqH AA concentrations. There were no significant correlations between AL and the AqH AA concentrations in either men or women.

**Table 3:** Correlation between AL and AqH AA concentrations.

	Men				Women			
	r_P_	p-value	partial.r*	p-value	r_P_	p-value	partial.r*	p-value
AqH AA concentrations	0.045	0.714	0.082	0.508	0.151	0.144	0.032	0.760

AL, axial length; AqH, aqueous humour; AA, ascorbic acid; r_p_, Pearson’s correlation coefficient. *Age and plasma AA concentration-adjusted Pearson’s correlation coefficient between AL and AqH AA concentrations.

### Correlation between ACD and AqH AA concentrations

Table 4 shows the sex-specific correlation coefficients between ACD and AqH AA concentrations. In women, ACD was positively correlated with AqH AA concentrations (partial.r = 0.275, P = 0.009) after adjustment. There were no significant correlations in men (partial.r = 0.049, P = 0.700).

**Table 4:** Correlation between ACD and AqH AA concentrations.

	Men				Women			
	r_P_	p-value	partial.r*	p-value	r_P_	p-value	partial.r*	p-value
AqH AA concentrations	−0.043	0.728	0.049	0.700	0.339	<0.001	0.275	0.009

ACD, anterior chamber depth; AqH, aqueous humour; AA, ascorbic acid; r_p_, Pearson’s correlation coefficient. *Age and plasma AA concentration-adjusted Pearson’s correlation coefficient between ACD and AqH AA concentrations.

## Discussion

The present study showed that AqH AA concentrations were significantly and positively correlated with ACD in women after adjusting for confounding factors (age and plasma AA concentrations).

The AA concentration in the corneal epithelium is the highest among all known tissue concentrations in the eye^4,24,25^. Since blood vessels are not distributed on the cornea, the cornea receives AA from the tears and the AqH^11,24,25^. In the present study, AA concentrations in the AqH were 1535 ± 326 μmol/L in men and 1733 ± 355 μmol/L in women, at levels similar to those reported by a US study with patients with cataract (1410 ± 550 μmol/L in men and 1640 ± 580 μmol/L in women)^3^. In contrast, Senthilkumari *et al*.^1^ reported that AqH AA concentrations were 1010 ± 469 μmol/L in men and 1138 ± 613 μmol/L in women with poor nutritional status in India. In this study, the plasma and AqH AA concentrations tended to be lower in men. Some studies have shown a similar trend^1,3^. Even considering the influence of dietary intake and preferences between the sexes, renal excretion is higher in men than in women^26^. Therefore, we speculate that the results were influenced by sex differences in excretion, absorption, and retention of AA. AA is considered to be actively transported from the blood through the ciliary body into the AqH^27,28^. However, the mean concentration ratio between AqH AA and plasma AA varied from 18 to 71 in previous studies^1–3^. Our study showed that the ratio was 32 in men and 33 in women. AqH AA concentrations reportedly have a positive correlation with plasma AA concentrations^1–3^. There was a positive correlation between plasma and AqH AA concentrations both in men (r = 0.420, P < 0.001) and in women (r = 0.316, P = 0.002) in this study, in support of past reports. In addition, Čanadanović *et al*.^29^ reported that AqH AA concentration decreases with age. Therefore, in order to investigate the correlation between ACD and AqH AA concentrations, we adjusted for two confounding factors (age and plasma AA concentrations).

Several *in vitro* and *in vivo* studies have shown that AA scavenges free radicals in phacoemulsification and reduces corneal endothelial-cell damage^12,13^. This protective effect on corneal endothelial cells is attributable to AA directly eliminating free radicals generated in the AqH. Therefore, as phacoemulsification always replaces the anterior chamber with irrigating solutions, AqH AA concentrations before surgery may have little involvement in radical scavenging during surgery. However, since corneal endothelial cells have the ability to absorb AA^30^, AA may act as a protective factor against oxidative stress even intracellularly^31^. Yue *et al*.^32^ and Reddy *et al*.^33^ reported that AA might be an important factor in endothelial-cell healing, migration, and regeneration. Moreover, Biaggi *et al*.^34^ reported that after phacoemulsification in dogs, AqH AA concentrations were reduced until 15 days postoperatively. Consequently, in patients with low AA concentrations in the AqH, corneal endothelial cells may be affected by oxidative damage from early postoperatively and extending to the long term. Because this was a cross-sectional study, we could not demonstrate the accelerated reduction of corneal endothelial cells in relation to AqH AA concentrations; there is a need for a prospective longitudinal study on the effects of AqH AA concentrations on long-term corneal endothelial-cell loss after phacoemulsification.

In this study, no correlation was found between AL and AqH AA concentrations (partial.r = 0.032, P = 0.760), but in women there was a positive correlation between ACD and AqH AA concentrations (partial.r = 0.275, P = 0.009), which remained even after adjusting for age and plasma AA concentrations. Therefore, the fact that AqH AA concentrations are lower in women with short ACD may suggest that corneal endothelial cells are more susceptible to postoperative oxidative damage.

There has been no report so far on the association between ACD and AqH AA concentrations. AqH AA concentrations could be low in women with short ACD due to low transportation capacity of AA into the AqH. Recently, Ma *et al*.^35^ reported that in the human ciliary epithelium sodium-dependent AA transporter (SVCT) 2 is expressed only in the pigmented epithelium, and glucose transporter (GLUT) 1 is predominately expressed in the nonpigmented epithelium. This may explain why SVCT2 and GLUT1 are involved in the maintenance of higher AqH AA concentrations in humans. Further, Senthilkumari *et al*.^1^ reported that polymorphisms in the SVCT genes encoding SVCT1 and SVCT2 influenced AqH AA concentrations. Although the relationship between the ACD and SVCT genes needs to be confirmed in future studies, women with short ACD may have a genotype that lowers AqH AA concentrations.

A strength of this study was that AA was evaluated using high-performance liquid chromatography (HPLC)-electrochemical detection systems. HPLC-electrochemical detection systems have high sensitivity and specificity for AA analysis^36^. In humans, few studies on AqH AA concentrations have employed HPLC-electrochemical detection systems.

This study had some limitations. We did not measure dietary AA intake in the present study. Variation in dietary intake and the time interval between the collection of blood and AqH samples may have introduced bias. The time interval between the collection of blood and AqH samples was approximately 1 month or less. However, in both cases, we collected samples after the patients had fasted for at least 5 h in order to ensure uniformity in the collection conditions. Hence, dietary variation may have been negligible. Moreover, in this report, both AqH and plasma AA concentrations were at levels comparable to those reported by previous studies in Japanese, American, and European populations^37–39^. Further, patients receiving dialysis or with eating disorders, dementia, and systemic inflammatory diseases were excluded from this study; therefore, we did not include patients with conditions causing markedly-poor nutritional status. Another limitation of this study is that we could not determine whether short ACD directly caused decrease in AqH AA concentrations. The ACD is affected by other factors such as lens vault, zonular weakness, iris curvature, and iris thickness in addition to the AL^19–21,40–43^. Changes in the lens and the morphology of the iris may also affect AqH AA concentrations. Therefore, we plan to further investigate the relationship between lens vault, iris curvature, iris thickness, intraocular pressure (IOP), and AqH AA concentrations in the future.

In conclusion, there was a positive correlation between ACD and AqH AA concentrations in women, and AqH AA concentrations were lower in women with short ACD. This may suggest that women with short ACD have low reducing power in the AqH and could be more susceptible to oxidative damage.

## Methods

This cross-sectional consecutive study was performed in accordance with the Declaration of Helsinki. It was approved by the institutional ethics review board of Dokkyo University Hospital (I-15-51). Informed consent was obtained from all participants.

### Patients

**Figure 1:**
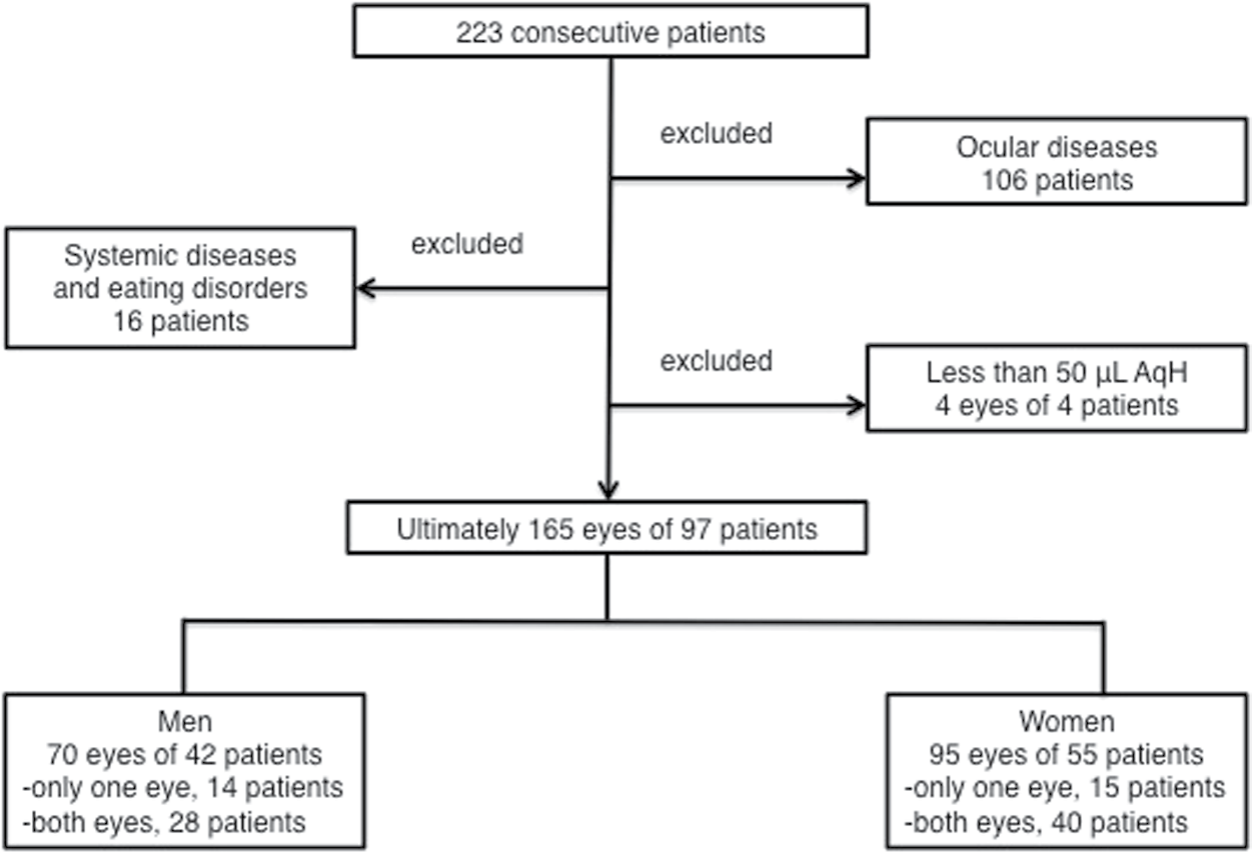
Patients with inherited cataract or trauma-related cataract, prior intraocular surgeries, prior laser treatment, congenital eye disease, corneal disease, acute infection, uveitis, acute angle closure glaucoma, primary angle closure glaucoma, primary open angle glaucoma, retinal disease, exfoliation syndrome, renal failure, eating disorders, dementia, and inflammatory systemic diseases were excluded from the study, as were those from whom we could not obtain more than 50 μL of AqH due to a shallow anterior chamber. Ultimately, 165 eyes of 97 patients were included.

A total of 223 consecutive patients who visited Dokkyo Medical Hospital and other associated hospitals to undergo small incision cataract surgery from April 2017 to January 2018 were recruited. Patients with inherited cataract or trauma-related cataract, prior intraocular surgeries, prior laser treatment, congenital eye disease, corneal disease, acute infection, uveitis, acute angle closure glaucoma, primary angle closure glaucoma, primary open angle glaucoma, retinal disease, exfoliation syndrome, renal failure, eating disorders, dementia, and inflammatory systemic diseases were excluded from the study, as were those from whom we could not obtain more than 50 μL of AqH due to a shallow anterior chamber. Ultimately, 165 eyes of 97 patients were included. The included patients had normal IOP (defined as lower than 21 mm Hg) and were not using any topical or internal intraocular tension depressors. Moreover, we did not use capsule stabilisation devices or intraocular lens scleral suture fixation in the patients. The patient selection procedure and distribution of the study population are shown in Fig. 1.

### Clinical examination

All subjects underwent a thorough ophthalmic evaluation before cataract surgery. Uncorrected visual acuity (UCVA) and best-corrected visual acuity (BCVA) were tested with Landolt C charts. IOP was measured with a non-contact tonometer (TONOREFII; Nidek Corp, Gamagori, Japan). Lens NS was graded with a slit lamp using the Emery-Little classification with scores ranging from 1 to 5. Corneal ECD was measured using specular microscopy (Nonconrobo FA-3509; Konan Medical, Hyogo, Japan). AL, ACD, and CCT were obtained using partial optical coherence interferometry (IOLMaster; Carl Zeiss Meditec AG, Jena, Germany).

### AqH and blood samples

The sampling of AqH was performed by the surgeon who performed the cataract surgery. Patients undergoing surgery in both eyes may select a 5- or 7-day interval between the operations. The AqH was obtained under sterile conditions at the beginning of surgery after topical anaesthesia. First, the AqH was obtained by directly puncturing the corneal limbus with a 30-gauge needle attached to a disposable tuberculin syringe without touching the iris, lens, or corneal endothelium. An AqH sample of at least 50 μL was obtained from the periphery of the anterior chamber. Immediately after collection, the AqH was frozen at −20 °C using a cooling system (Corning® CoolBox™ M30 System; Corning, NY, USA) and transferred to the laboratory. The AqH samples were added to cold 10% metaphosphoric acid (MPA) and centrifuged at 15000 rpm for 15 min at 4 °C. The 50-μL samples were collected and stored at −80 °C until AA could be measured. The blood samples were drawn into collection tubes (Terumo Corporation, Tokyo, Japan) containing ethylenediaminetetraacetic acid (EDTA)−2Na as anticoagulant and centrifuged at 3000 rpm for 10 min at 4 °C. After centrifugation, 500-μL plasma was added at the exact same volume of cold 10% MPA and centrifuged at 15000 rpm for 15 min at 4 °C. Then, the 500-μL supernatant was stored at −80 °C until use. Both AqH and blood samples were collected at least 5 h after the last meal.

### Determination of AA

**Figure 2:**
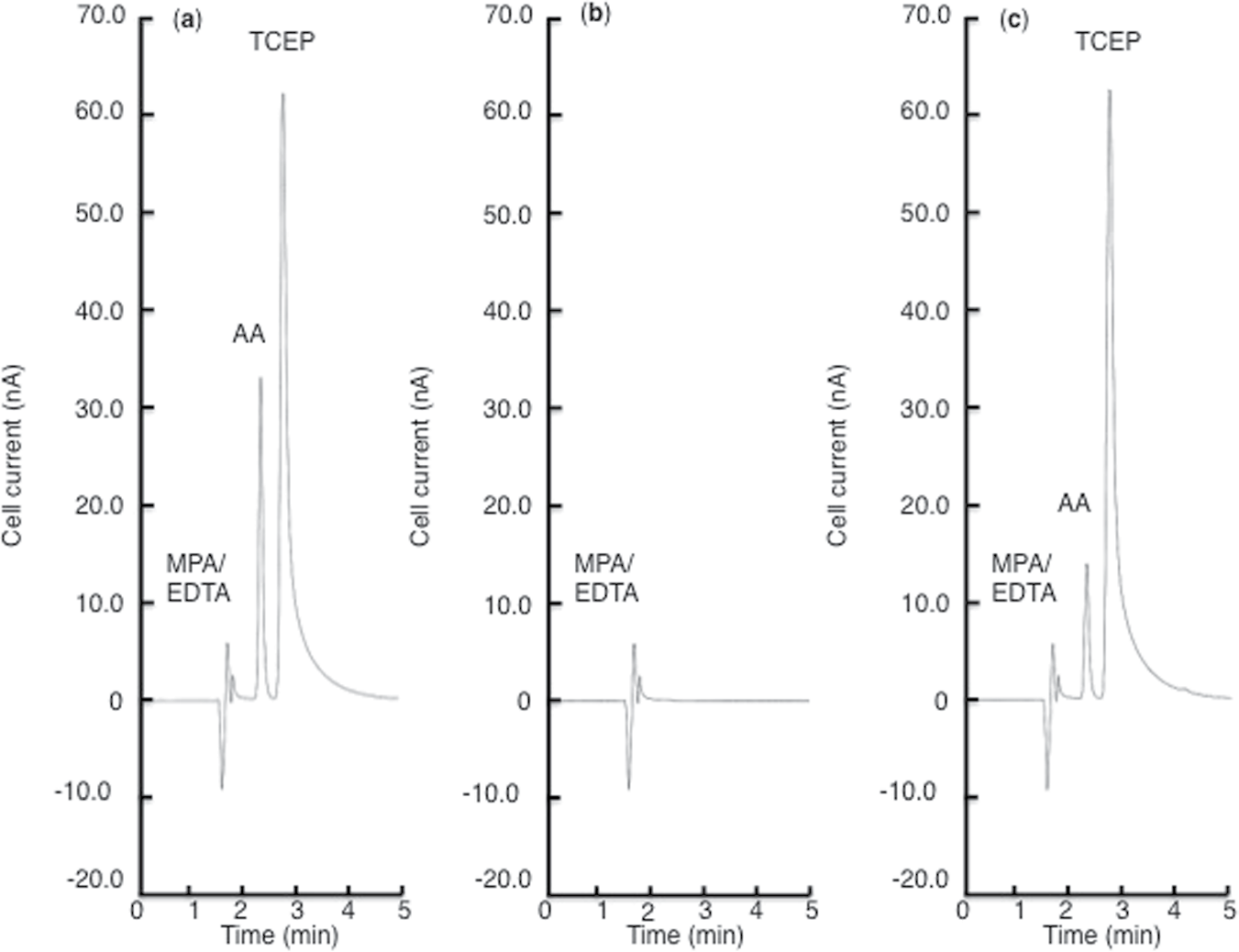
Representative high-performance liquid chromatography -electrochemical detection chromatograms of AA, metaphosphoric acid (MPA)/ethylenediaminetetraacetic acid (EDTA), and tris (2-carboxyethyl) phosphine hydrochloride (TCEP). (**A**) AA standard solution (5.7 μM) in 5% MPA/EDTA reduced by 35mM TCEP. (**B**) 5% MPA/EDTA. (**C**) Aqueous humour sample in 5% MPA/EDTA reduced by 35 mM TCEP for 2 h on ice.

AA was analysed using an HPLC-electrochemical detection method. The samples were treated as previously reported^36^. Detection was performed with a Waters 2695 separations module coupled with a Waters 2465 electrochemical detector (Nihon Waters, Tokyo, Japan). After thawing, the samples were reduced with 35 mM tris (2-carboxyethyl) phosphine hydrochloride for 2 h on ice. After reduction, the reaction mixture was analysed for total AA with an HPLC-electrochemical detection method. Separation was performed on an Atlantis dC18 5-μm column (4.6 × 150 mm) combined with an Atlantis dC18 5-μm guard column (4.6 × 20 mm) (Nihon Waters, Tokyo, Japan). The mobile phase comprised 50 mM phosphate buffer (pH 2.8), 540 μM EDTA, and 2% methanol. The flow rate was 1.3 mL/min, and electrical signals were recorded using an electrochemical detector with a glassy carbon electrode at +0.6 V. Representative HPLC-electrochemical detection chromatograms are shown in Fig. 2.

### Statistical analyses

Statistical analyses were performed using software (SPSS 24.0; IBM Corp., Armonk, NY, USA). The data are expressed as mean ± SD. A P value less than 0.05 was considered statistically significant. The normality of data distribution was tested with histograms and the Shapiro-Wilk test. Categorical data were assessed using the Mann-Whitney U test, and continuous variables were assessed using independent Student’s t-tests. Pearson’s correlation coefficient was calculated for normally-distributed data. If the data distribution was not normal, Spearman correlation analyses were used. To examine the relationship between ACD and AqH AA concentration, partial correlation coefficients were calculated for statistical adjustment of covariates (age and plasma AA concentration).

## Data Availability

The corresponding author had full access to all the data in the study and all authors shared final responsibility for the decision to submit for publication.
